# LncRNAs and their regulatory networks in breast muscle tissue of Chinese Gushi chickens during late postnatal development

**DOI:** 10.1186/s12864-020-07356-6

**Published:** 2021-01-09

**Authors:** Yuanfang Li, Wenjiao Jin, Bin Zhai, Yi Chen, Guoxi Li, Yanhua Zhang, Yujie Guo, Guirong Sun, Ruili Han, Zhuanjian Li, Hong Li, Yadong Tian, Xiaojun Liu, Xiangtao Kang

**Affiliations:** 1 College of Animal Science and Veterinary Medicine, Henan Agricultural University, Zhengzhou, 450002 China; 2 Henan Innovative Engineering Research Center of Poultry Germplasm Resource, Henan Agricultural University, Zhengzhou, 450002 China

**Keywords:** Chicken, Breast muscle, lncRNAs, Regulatory network, ceRNA

## Abstract

**Background:**

Chicken skeletal muscle is an important economic product. The late stages of chicken development constitute the main period that affects meat production. LncRNAs play important roles in controlling the epigenetic process of growth and development. However, studies on the role of lncRNAs in the late stages of chicken breast muscle development are still lacking. In this study, to investigate the expression characteristics of lncRNAs during chicken muscle development, 12 cDNA libraries were constructed from Gushi chicken breast muscle samples from 6-, 14-, 22-, and 30-week-old chickens.

**Results:**

A total of 1252 new lncRNAs and 1376 annotated lncRNAs were identified. Furthermore, 53, 61, 50, 153, 117, and 78 DE-lncRNAs were found in the *W14* vs. *W6, W22* vs. *W14, W22* vs. *W6, W30* vs. *W6, W30* vs. *W14*, and *W30* vs. *W22* comparison groups, respectively. After GO enrichment analysis of the DE-lncRNAs, several muscle development-related GO terms were found in the *W22* vs. *W14* comparison group. Moreover, it was found that the MAPK signaling pathway was one of the most frequently enriched pathways in the different comparison groups. In addition, 12 common target DE-miRNAs of DE-lncRNAs were found in different comparison groups, some of which were muscle-specific miRNAs, such as gga-miR-206, gga-miR-1a-3p, and miR-133a-3p. Interestingly, the precursors of four newly identified miRNAs were found to be homologous to lncRNAs. Additionally, we found some ceRNA networks associated with muscle development-related GO terms. For example, the ceRNA networks contained the *DYNLL2* gene with 12 lncRNAs that targeted 2 miRNAs. We also constructed PPI networks, such as *IGF-I*-*EGF* and *FZD6*-*WNT11*.

**Conclusions:**

This study revealed, for the first time, the dynamic changes in lncRNA expression in Gushi chicken breast muscle at different periods and revealed that the MAPK signaling pathway plays a vital role in muscle development. Furthermore, *MEF2C* and its target lncRNA may be involved in muscle regulation through the MAPK signaling pathway. This research provided valuable resources for elucidating posttranscriptional regulatory mechanisms to promote the development of chicken breast muscles after hatching.

**Supplementary Information:**

The online version contains supplementary material available at 10.1186/s12864-020-07356-6.

## Background

Muscle, especially skeletal muscle, is an important part of an animal [1]. In livestock production, skeletal muscle is an important economic product for human consumption. After birth, muscle weight continuously increases, and the growth of skeletal muscle is mainly achieved by increasing the hypertrophy of existing muscle fibers [2]. Compared with the embryonic stage, from hatching to marketing or elimination (referred to as late postnatal development), the development of chickens at this stage is an important period affecting meat production [3]; therefore, it is essential to study skeletal muscle growth and development during the postnatal late development of important agricultural species.

Muscle development is a complex multistage process in which many genes cooperate to regulate each stage [4]. Several candidate genes, such as the growth hormone secretagogue receptor (*GHSR*) [5], insulin-like growth factors (*IGFs*) [6], transforming growth factor beta 2 (*TGFβ2*) [7], and myocyte enhancer factor 2B (*MEF2B*) [8], have been identified to play important roles in the growth of chickens. Although many genes play important roles in chicken muscle growth, studies have shown that only a small percentage (1–2%) of the genome encodes proteins in mammals, and tens of thousands of intergenic sites are transcribed into noncoding RNA [9]. In the past few years, regulatory RNAs, such as miRNAs, piRNAs, snoRNAs, and long noncoding RNAs (lncRNAs), have appeared to play roles in many important biological processes [10]. In complex organisms, lncRNAs contain hidden regulatory information that can play a role in the regulation of gene expression [11]; therefore, lncRNAs are important molecules that can affect the growth and development of chicken skeletal muscle.

LncRNAs are a class of non-protein-coding transcripts that are more than 200 bp in length [12]. Several lncRNAs have been shown to be expressed during development and have been shown to play an important role in epigenetic processes that control differentiation and development. For example, as one of the earliest identified lncRNAs, H19 also plays a regulatory role in various growth and development processes [13]. *MUNC* is a lncRNA that promotes skeletal muscle production by stimulating the adjacent myogenic differentiation antigen (*MyoD*) gene in C2C12 cells [14]. A novel lncRNA, *Irm*, has been shown to interact with *MEF2D* to enhance myogenic differentiation [15]. LncRNAs can exert *cis*-regulatory effects in biological processes. For instance, it has been indicated that lncRNA-Six1 cis regulates the *Six1* gene and encodes a micropeptide to activate *Six1*, thereby promoting cell proliferation and participating in muscle growth [16]. In addition, lncRNAs can also play a *trans*-regulatory role in biological processes. For example, the noncoding RNA *H19* can be used as a trans-regulator of *IGF2* [17]. Moreover, lncRNAs can also act as ceRNAs to protect mRNAs and act as a molecular sponge to inhibit miRNA targeting of mRNAs. For example, *lncIRS1* acts as a sponge of the miR-15 family, regulating the expression of insulin receptor substrate 1 (*IRS1*), thereby promoting skeletal muscle production [18]. Although an increasing number of lncRNAs have been characterized by high-throughput sequencing studies, there are few reports on the regulation of lncRNAs in chicken muscle development. Therefore, it is important to study the expression characteristics of lncRNAs during chicken muscle development.

Gushi chicken is an excellent variety of egg- and meat-providing chicken native to Gushi County, Henan Province, China. Gushi chicken is tender and delicious, with a fresh and unique flavor, and eliminated hens are often used as meat. Although Gushi chickens have many excellent characteristics, their growth rate is slightly slower than that of commercial broilers. Our previous histological study of this type of breast muscle showed that before 22 weeks of age, muscle fiber diameter grew rapidly, and after 22 weeks of age, the relationship between the diameter and density of the breast muscle fibers remained balanced [19]. To understand and control the growth and development of Gushi chicken skeletal muscle, we must understand the molecular regulation mechanism of different stages of skeletal muscle development. In this study, we identified lncRNAs by deep-sequencing data from four different stages (6, 14, 22, and 30 weeks) of Gushi chicken skeletal muscle development. Differentially expressed lncRNAs were used to predict *cis*- and *trans*-target genes to construct potential lncRNA-mRNA interaction networks and explore important signaling pathways. Then, the lncRNA data were combined with mRNA and miRNA data to construct potential lncRNA-miRNA-mRNA interaction networks and explore the regulatory networks that play a role in chicken skeletal muscle development. In summary, this study identifies differentially expressed lncRNAs at different stages of postnatal late developmental and provides predictions about the associated interaction networks, which can be used to further study the molecular regulation mechanism of chicken muscle development.

## Results

### Identification and characterization of lncRNAs

**Fig. 1:**
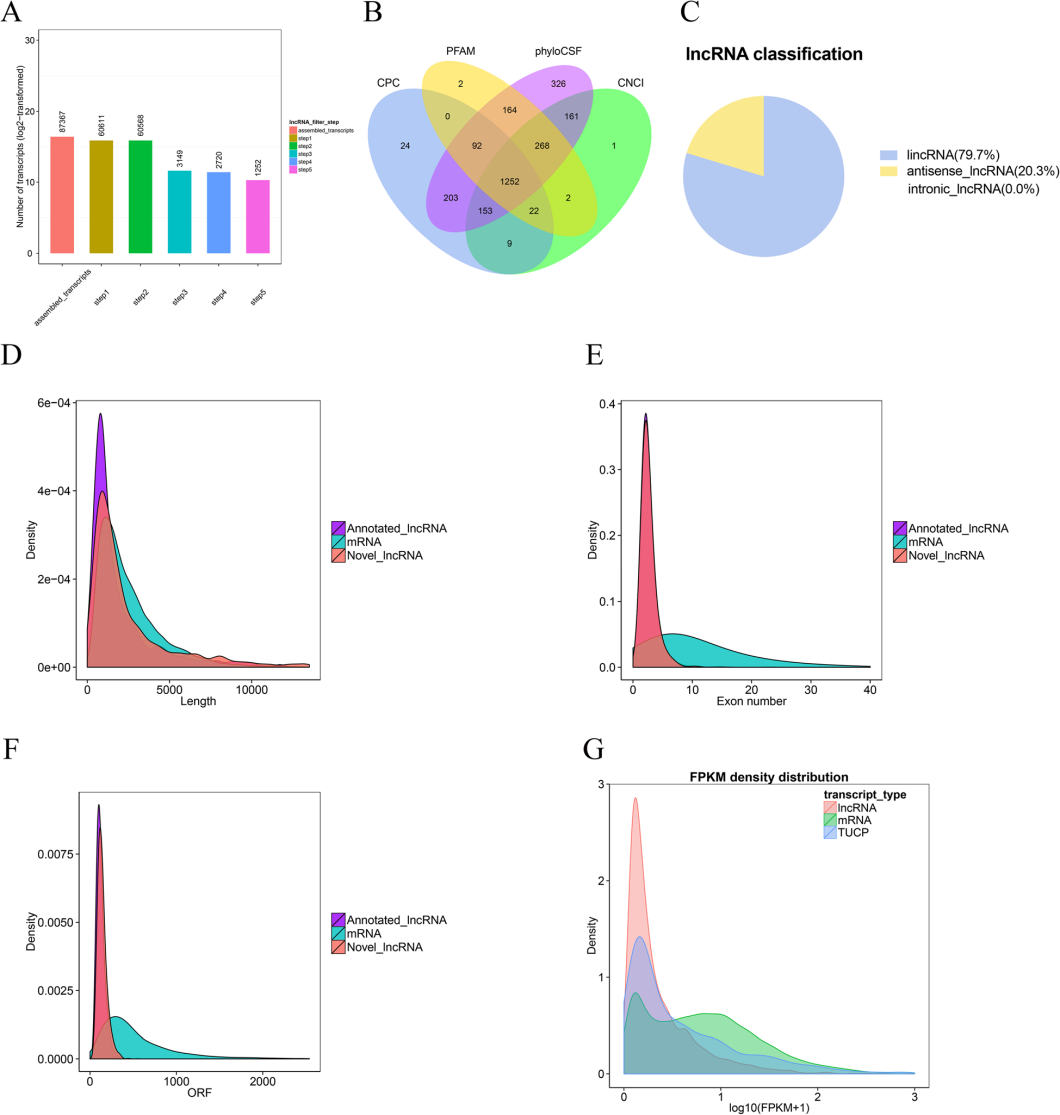
Characterization of lncRNAs. **a** Workflow used to define and identify the novel and annotated lncRNAs. **b** LncRNA identification through four databases, namely, Coding Potential Calculator (CPC), protein families database (PFAM), phylogenetic codon substitution frequency (PhyloCSF) and Coding-Noncoding Index (CNCI); (**c**) The distribution of lncRNA classification. **d**, **e**, **f** Distribution of transcript lengths, distribution of the number of exons per transcript, and distribution of the number of ORFs (mRNA: green, annotated lncRNA: purple, and novel lncRNAs: red). **g** Transcript expression levels (mRNA: green, lncRNA: red, and TUCP: purple)

Based on the Illumina HiSeq 2500 platform, a minimum of 89,496,872 raw reads were obtained from each library, with a clean base ranging from 12.75 Gb to 16.66 Gb and an error rate of 0.01 or 0.02 (Table S1). To generate a complete annotation of the noncoding transcriptome of the Gushi chicken breast muscle tissue beyond the currently annotated transcriptome, we first used Cuffmerge [20] to combine and then screen the transcripts from each sample. In this study, a total of 20,438 transcripts were identified, 16,342 of which were mRNAs. In addition, a total of 1376 lncRNAs had been previously annotated, 1252 novel lncRNAs were identified (Fig. 1a, b), and 1468 transcripts of uncertain coding potential (TUCPs) were screened. Only two types of lncRNAs were identified: an overwhelming majority of long intergenic noncoding RNAs (lincRNA) (79.7%) and a minority of antisense lncRNAs (20.3%) (Fig. 1c). LncRNAs in breast muscle tissue had a lower total transcript length, fewer exons, and fewer open reading frame (ORF) numbers than mRNAs (Fig. 1d-f) and a lower average transcript abundance (Fig. 1g).

### Characteristics of differentially expressed lncRNAs

**Fig. 2:**
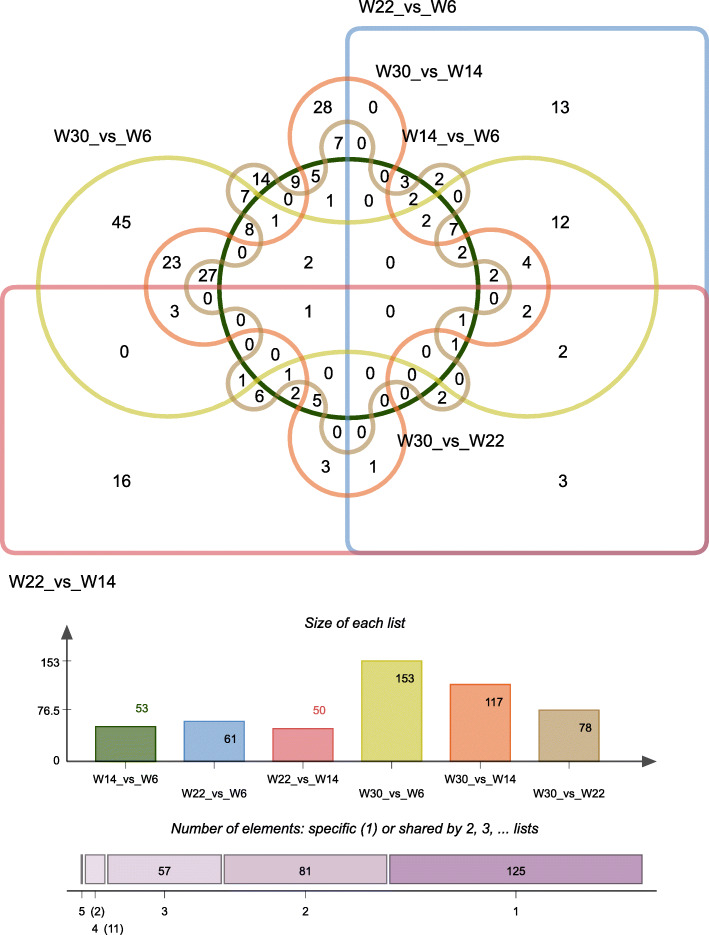
Venn diagram of DE-lncRNAs in six comparison groups

**Fig. 3:**
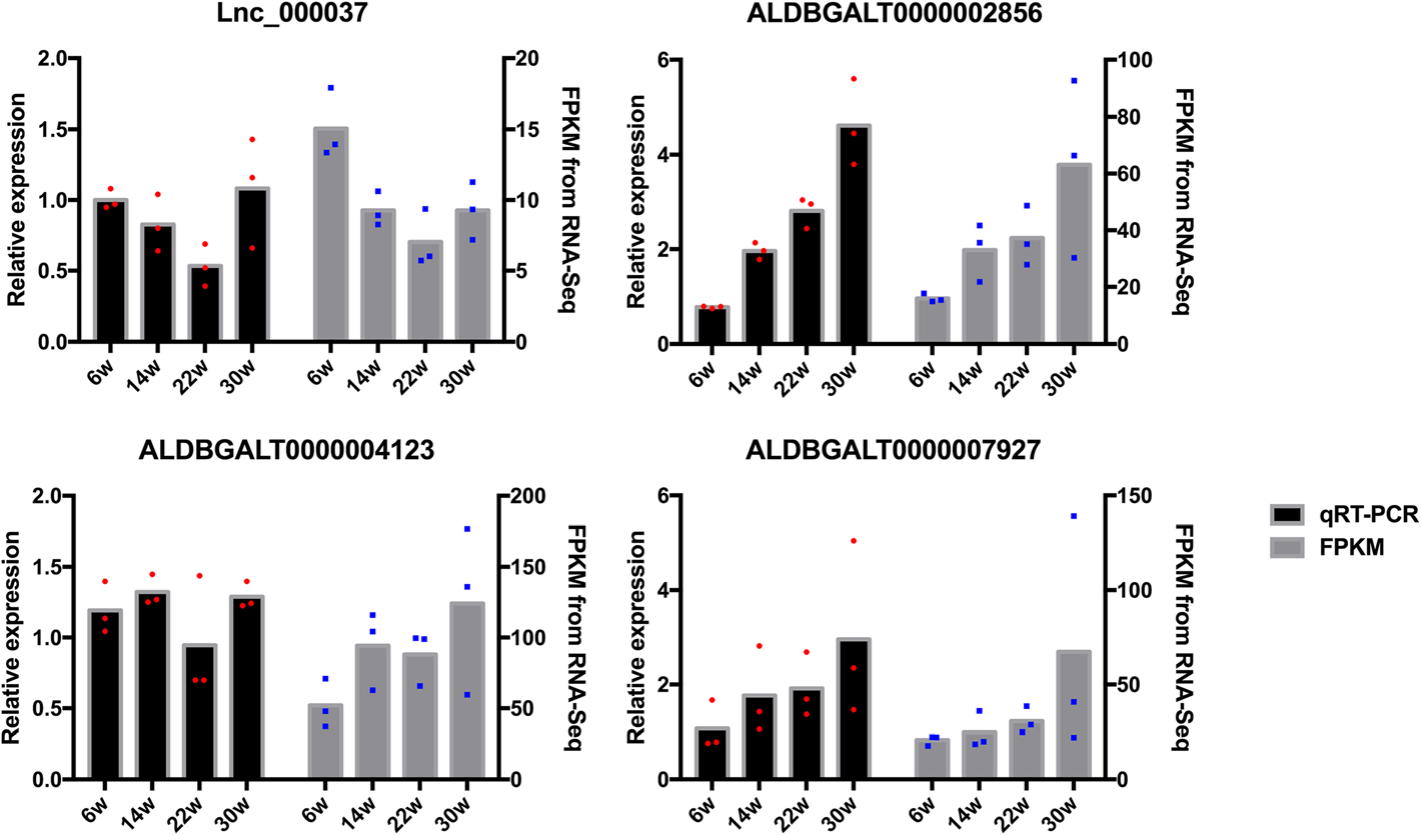
qRT-PCR validation of differentially expressed lncRNAs

To gain insight into the key lncRNAs involved in chicken breast muscle development, we analyzed the differentially expressed lncRNAs (DE-lncRNAs) (|fold change, FC|≥1.7, *q*-value < 0.05) at four different developmental stages, namely, 6 weeks (W6), 14 weeks (W14), 22 weeks (W22) and 30 weeks (W30). Among the six different comparison groups, there were 53, 61, 50, 153, 117, and 78 DE-lncRNAs in the *W14* vs. *W6, W22* vs. *W14, W22* vs. *W6, W30* vs. *W6, W30* vs. *W14*, and *W30* vs. *W22* comparison groups, respectively. Venn diagram analysis showed that there were no common DE-lncRNAs among the six comparison groups (Fig. 2). Only LNC_000920 was commonly found in the following five comparison groups: *W14* vs. *W6, W22* vs. *W14, W30* vs. *W6, W30* vs. *W14*, and *W30* vs. *W22.* Moreover, LNC_000255 appeared in the following comparison groups: *W14* vs. *W6, W22* vs. *W14, W22* vs. *W6, W30* vs. *W6,* and *W30* vs. *W14*. Then, the DE-lncRNAs were identified by a DEGseq (differentially expressed gene, DEG) analysis, and DE-lncRNAs were clustered based on their expression profiles (Fig. S1). The clustering results showed that the intragroup repeats of each group were clustered together, indicating that the intragroup differences were smaller than the intergroup differences, which proved that the data were reliable. In addition, we found that 22 weeks clustered close to 14 weeks, followed by 6 weeks, and the farthest distance was from 30 weeks. In addition, we also found that the number of common DE-lncRNAs between *W22* and *W14* was the lowest (Fig. 2). It is possible that from 14 weeks - 22 weeks, many of the same lncRNAs played a common role, which also led to the reduction in DE-lncRNA that was commonly seen in the six comparison groups. Moreover, after identifying DE-lncRNAs, we analyzed the chromosome distribution information of the DE-lncRNAs and found that DE-lncRNAs were distributed in almost all chromosomes but not in chromosomes 22 and 27, and the largest number was found in chromosome 1 (Fig. S2). In addition, we selected several lncRNAs for data validation. The lncRNA expression level was determined and showed a similar pattern to that of the RNA-seq data (Fig. 3), indicating that the RNA-seq data were authentic.

**Fig. 4:**
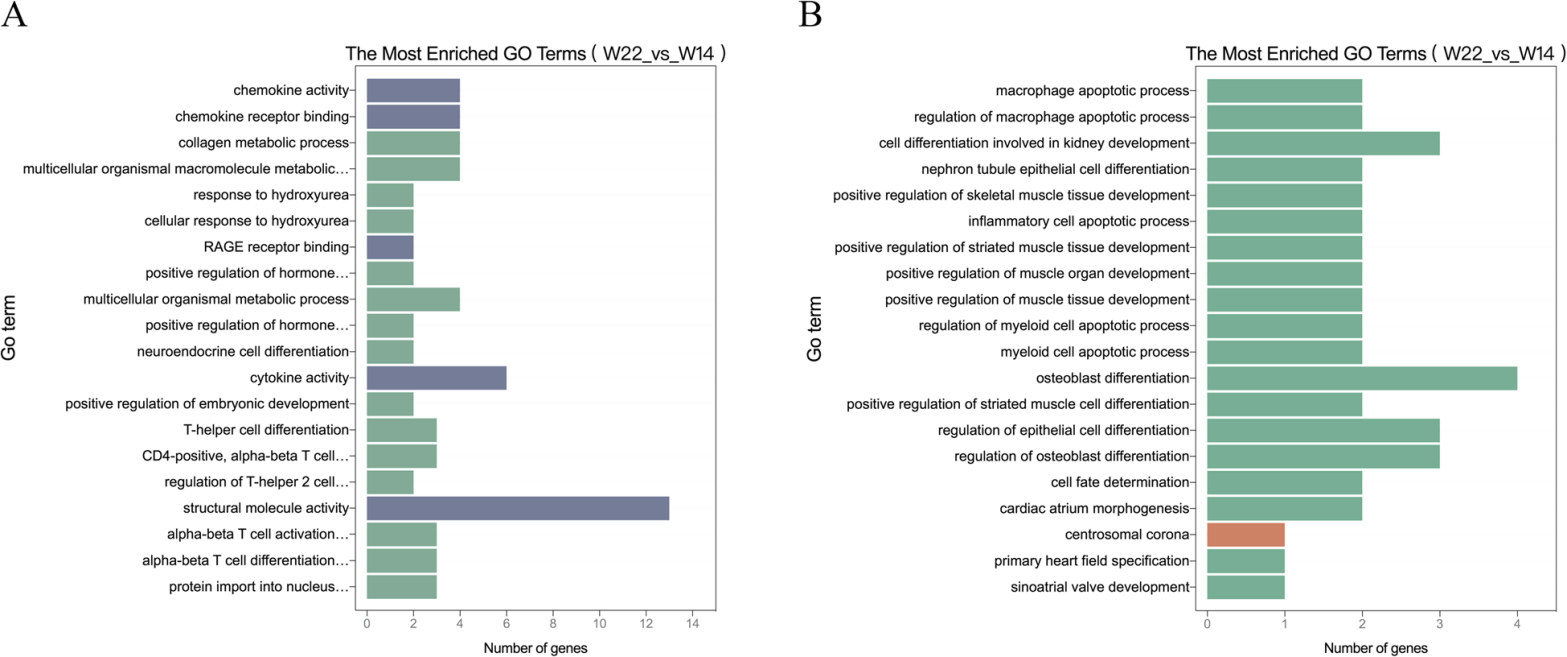
The enriched GO terms of the DE-lncRNA. **a** Cis-target genes in the *W22* vs. *W14* comparison groups. **b** Trans-target genes in the *W22* vs. *W14* comparison groups

To investigate the possible functions of the DE-lncRNAs in breast muscle between the different developmental stages, we conducted Gene Ontology (GO, http://www.geneontology.org/) enrichment analysis to uncover the enriched biological process terms associated with DE-lncRNA-targeted DEGs for each comparison group. Only the top 20 GO terms for the *W14* vs. *W6*, *W22* vs. *W14*, and *W30* vs. *W22* comparison groups are shown in Fig. 4 and Fig. S3. The *cis*-targets of all lncRNAs were predicted with a 100-kb upstream and downstream range. The GO enrichment analysis of the *cis*-targets of lncRNAs showed that only one growth- and development-related GO term, called positive regulation of embryonic development, was found in the *W22* vs. *W14* comparison group (Fig. 4a). In addition, we predicted the regulation in *trans* between lncRNAs and genes by the Pearson correlation coefficient r > 0.95. In the GO analysis of the *trans*-targets of lncRNAs, we found several muscle development-related GO terms only in the *W22* vs. *W14* comparison group (Fig. 4b), such as positive regulation of skeletal muscle tissue development, positive regulation of striated muscle tissue development, positive regulation of muscle organ development, positive regulation of muscle tissue development, and positive regulation of striated muscle cell differentiation.

**Fig. 5:**
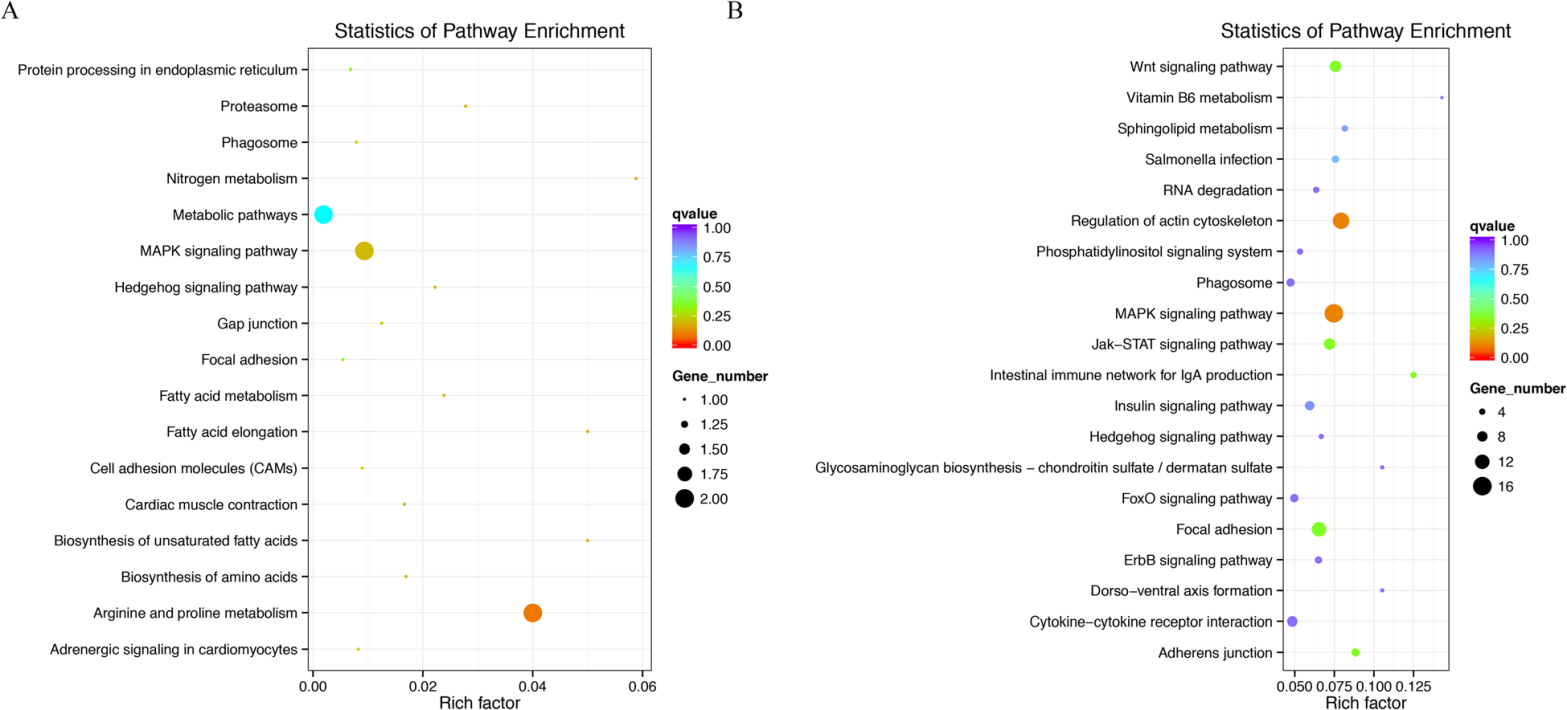
The enriched KEGG pathways of the DE-lncRNA. **a** Trans-target genes in the *W22* vs. *W14* comparison groups. **b** Trans-target genes in the *W30* vs. *W22* comparison groups

To further understand how DE-lncRNA-targeted DEGs play roles in regulating chicken muscle development, we performed Kyoto Encyclopedia of Genes and Genomes (KEGG, http://www.genome.jp/kegg/) pathway analysis for each comparison. In the KEGG pathway analysis of the *cis*-targets of lncRNAs, the phagosome pathway was identified as the most significantly enriched pathway for the *W14* vs. *W6* comparison group (Fig. S4A). Furthermore, for the *W22* vs. *W14* comparison group, the endocytosis pathway was identified as the most significantly enriched pathway (Fig. S4B). Additionally, the focal adhesion and cytokine-cytokine receptor interaction pathways were the top two pathways for the *W30* vs. *W22* comparison group (Fig. S4C). Moreover, the KEGG pathway analysis of the *trans*-targets of lncRNAs showed that the propanoate metabolism pathway and fatty acid metabolism pathway were the top two pathways for the *W14* vs. *W6* comparison group (Fig. S4D). In the *W22* vs. *W14* comparison group, the two top pathways were arginine and proline metabolism and the MAPK signaling pathway (Fig. 5a). In addition, for the *W30* vs. *W22* comparison group, there were two top pathway terms, the MAPK signaling pathway and the regulation of actin cytoskeleton pathway (Fig. 5b). We found that the MAPK signaling pathway was one of the most frequently enriched pathways in both the *W22* vs. *W14* and *W30* vs. *W22* comparison groups.

### Interactions between lncRNAs and mRNAs during breast muscle development

**Fig. 6:**
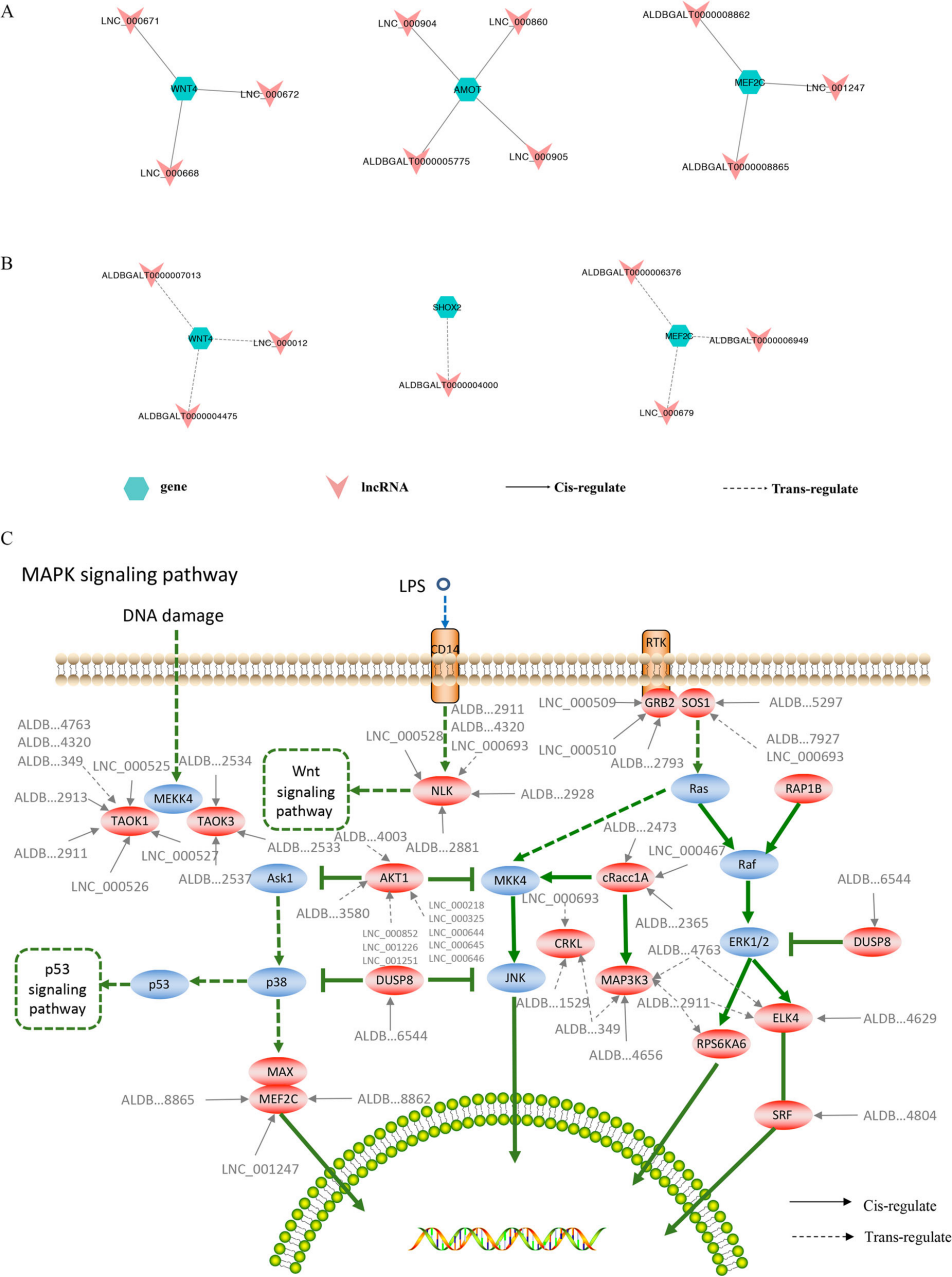
The cis- and trans-networks of DEGs and DE-lncRNAs of muscle development-related GO terms and signaling pathways. **a** The cis-target networks associated with enriched GO terms. **b** The trans-target networks associated with enriched GO terms. **c** The networks of cis- and trans-target DEGs of DE-lncRNAs (r> 0.98) in the MAPK signaling pathway (The network relations were refer to the KEGG website: https://www.genome.jp/kegg/pathway.html. Only the red nodes represent the DE lncRNA-targeted genes. Solid arrows represent cis-targets, and dashed arrows represent trans-targets)

To explore how lncRNAs interact with their target genes to regulate chicken muscle development and to identify key molecular players in the process, we first predicted the *cis*- and *trans*-targets of DE-lncRNAs and then constructed the regulatory networks between DE-lncRNAs and their target genes. A total of 13,460 *cis-*regulatory interaction relationships were detected between 2309 lncRNAs and 7783 mRNAs (Table S2). In addition, 13,343 *trans-*regulatory interaction relationships were detected between 733 lncRNAs and 2190 mRNAs (Table S3). Moreover, we constructed the lncRNA-mRNA networks of *cis-* and *trans-*targets for muscle development related to the top 20 GO terms, including positive regulation of embryonic development, positive regulation of skeletal muscle tissue development, positive regulation of striated muscle tissue development, positive regulation of muscle organ development, positive regulation of muscle tissue development and positive regulation of striated muscle cell differentiation. In the networks of *cis*-target DEGs of DE-lncRNAs of the muscle development-related GO terms, we found a total of 10 interaction relationships between 3 genes and 10 lncRNAs (Fig. 6a). In addition, in the networks of *trans*-target DEGs of DE-lncRNAs of the muscle development-related GO terms, we found 7 interaction networks between 3 genes and 7 lncRNAs (Fig. 6b). Furthermore, we also generated the lncRNA-mRNA networks of the frequently enriched MAPK signaling pathway, which had a total of 25 interaction relationships between 11 genes and 25 *cis-*regulating lncRNAs and 27 interaction relationships between 8 genes and 17 *trans-*regulating lncRNAs (Fig. 6c). Interestingly, we found that the networks containing *MEF2C* and its targeting lncRNAs (ALDBGALT0000008862, ALDBGALT0000008865, LNC_001247) were not only in the muscle development-related GO terms but also in the MAPK signaling pathway.

### LncRNA-miRNA interactions

**Fig. 7:**
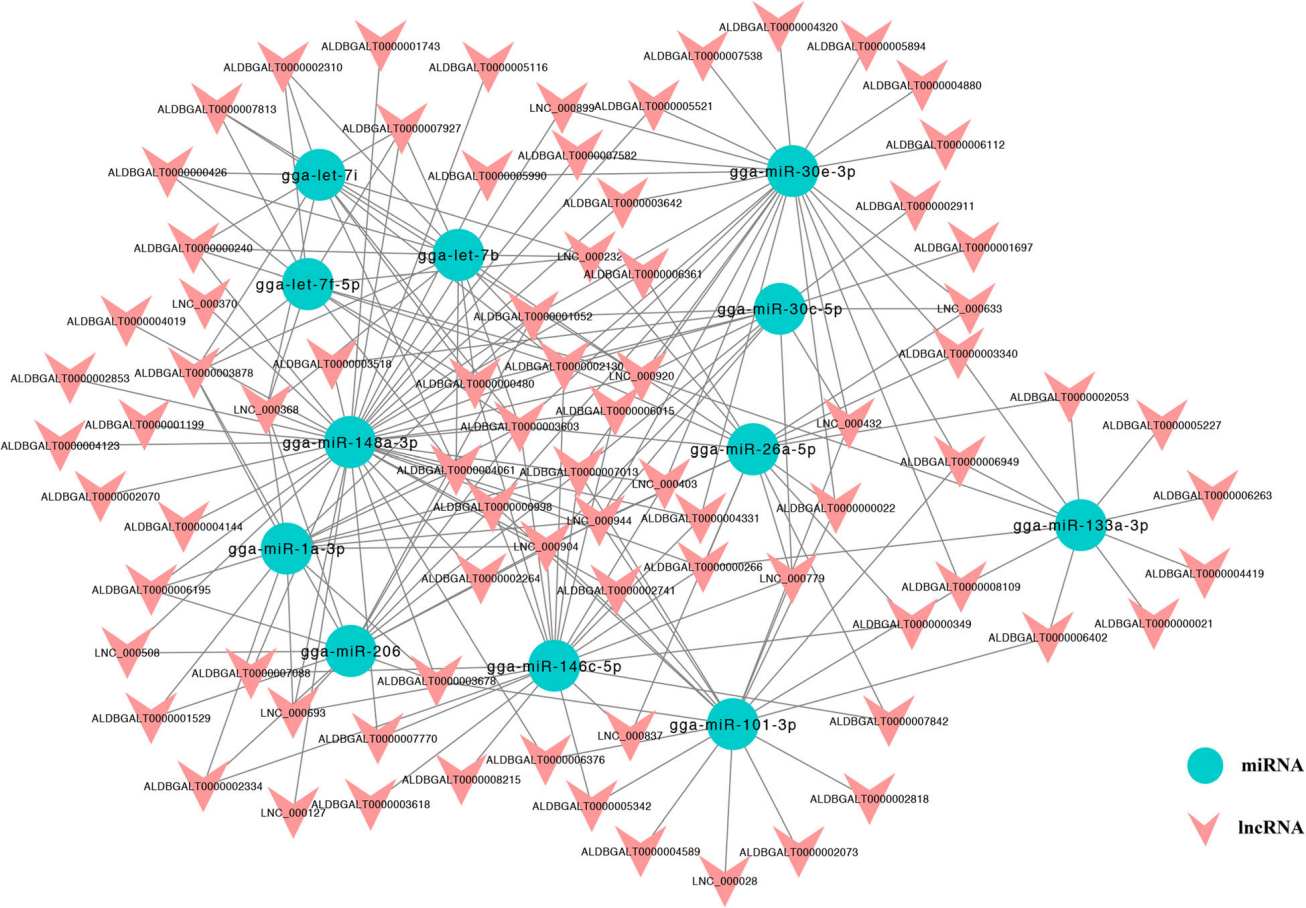
Interaction network between 12 common DE-miRNAs and their target lncRNAs of different comparison groups

**Fig. 8:**
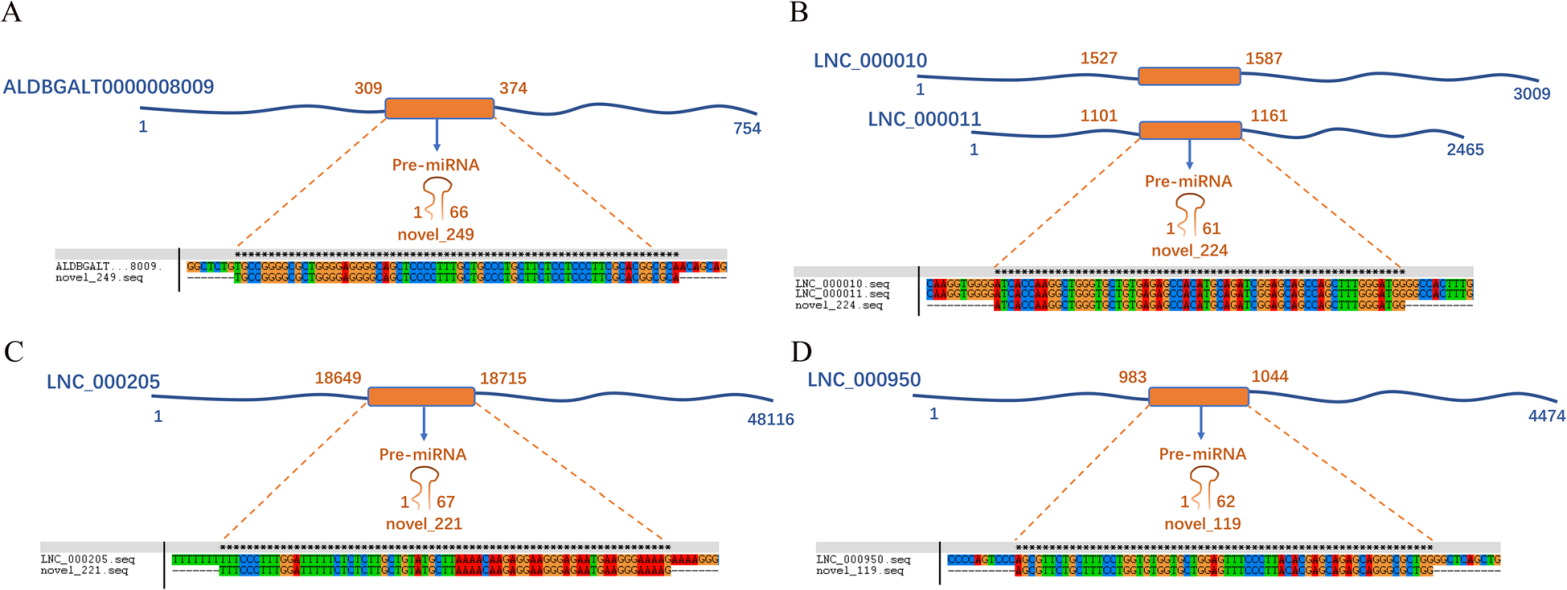
Homology of lncRNAs with pre-miRNAs. **a** Homology of ALDBGALT0000008009 with gga-miR-N1. **b** Homology of lnc_000010 and lnc_000011 with gga-miR-N2. **c** Homology of lnc_000205 with gga-miR-N3. **d** Homology of lnc_000950 with gga-miR-N4

In our previous study [19], we found 388 known miRNAs and 31 novel miRNAs. To explore the interactions between lncRNAs and miRNAs, we predicted the target relationship between lncRNAs and miRNAs in different comparison groups (Table S4). Twelve common target DE-miRNAs of DE-lncRNAs were found in different comparison groups. It is important that some of them were muscle-specific miRNAs, such as gga-miR-206, gga-miR-1a-3p, and miR-133a-3p. Only the lncRNAs (FPKM> 1) of these miRNA targets are shown in Fig. 7. Then, we predicted the pre-miRNAs with homology to lncRNAs. Unexpectedly, the precursors of four newly identified miRNAs were found to be homologous to lncRNAs, and the precursors were temporarily named gga-miR-N1, gga-miR-N2, gga-miR-N3 and gga-miR-N4 (Fig. 8, Table S5). For example, the pre-miRNA of gga-miR-N1 exactly matches ALDBGALT0000008009 at its position from 309 to 374, and the pre-miRNA of gga-miR-N2 exactly matches lnc_000010 at its position from 1527 to 1587, and it also matches lnc_000011 from 1101 to 1161. These lncRNAs may form miRNA precursors through intracellular shearing and then could be processed to generate specific miRNAs that regulate the expression of target genes.

### LncRNA-miRNA-mRNA regulatory networks

**Fig. 9:**
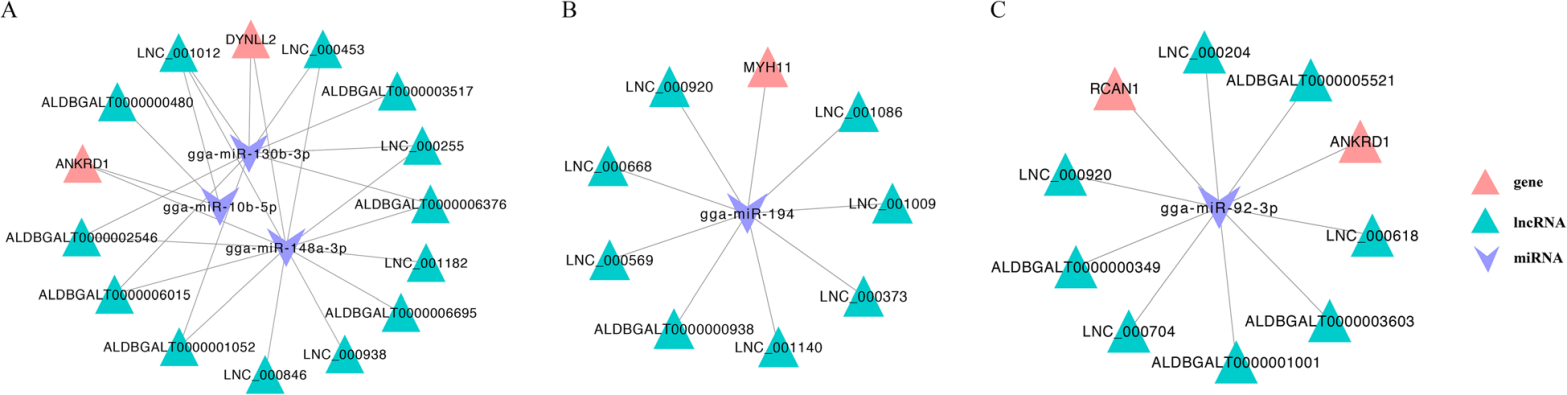
The lncRNA-miRNA-mRNA ceRNA networks of muscle development-related GO terms. **a** *W14* vs. *W6* comparison group; (**b**) *W22* vs. *W14* comparison group; (**c**) *W30* vs. *W22* comparison group

To identify potential ceRNA networks in the development of chicken breast muscle, we constructed ceRNA networks of DEGs, differentially expressed miRNAs (DEMs), and DE-lncRNAs (*q*-value < 0.05 and log2|FC|≧1) by Cytoscape (Fig. S5), and we found some ceRNA networks associated with muscle development-related GO terms (Fig. 9). For example, 445 ceRNA networks were found in the lncRNA-miRNA-mRNA network in the *W14* vs. *W6* comparison group (Fig. S5A). Among these networks, ankyrin repeat domain 1 (*ANKRD1*) is related to skeletal muscle cell differentiation, and it was involved in 13 ceRNA networks containing two miRNAs (miR-148a-3p and miR-10b-5p) and 12 lncRNAs (LNC_000846, ALDBGALT0000001052, LNC_000453, LNC_001182, ALDBGALT0000006695, ALDBGALT0000002546, ALDBGALT0000006376, LNC_000255, LNC_000938, ALDBGALT0000006015, LNC_001012, and ALDBGALT0000000480) (Fig. 9a). In addition, dynein light chain 2 (*DYNLL2*) is related to the myosin complex GO term, which was involved in 19 ceRNA networks with two miRNAs (gga-miR-148a-3p and gga-miR-130b-3p) and 12 lncRNAs (LNC_000846, ALDBGALT0000001052, LNC_000453, LNC_001182, ALDBGALT0000006695, ALDBGALT0000002546, ALDBGALT0000006376, LNC_000255, LNC_001012, LNC_000938, ALDBGALT0000006015, and ALDBGALT0000003517) (Fig. 9a). In the *W22* vs. *W14* comparison group, there were 76 ceRNA networks (Fig. S5B). Among them, myosin heavy polypeptide 11 (*MYH11*) is related to muscle cell differentiation, which was involved in 8 ceRNA networks containing gga-miR-194 and 8 lncRNAs (LNC_000668, LNC_000569, LNC_001009, ALDBGALT0000000938, LNC_001086, LNC_000373, LNC_000920, and LNC_001140) (Fig. 9b). Moreover, there were 450 ceRNA networks in the *W30* vs. *W22* comparison group (Fig. S5C). The skeletal muscle fiber development-related gene regulators of calcineurin 1 (*RCAN1*) and *ANKRD1* were involved in 8 ceRNA networks containing gga-miR-92-3p and 8 lncRNAs (LNC_000920, LNC_000704, ALDBGALT0000001001, ALDBGALT0000005521, LNC_000618, ALDBGALT0000000349, LNC_000204, and ALDBGALT0000003603) (Fig. 9c).

### Protein-protein interaction (PPI) network of DE-lncRNA target genes

**Fig. 10:**
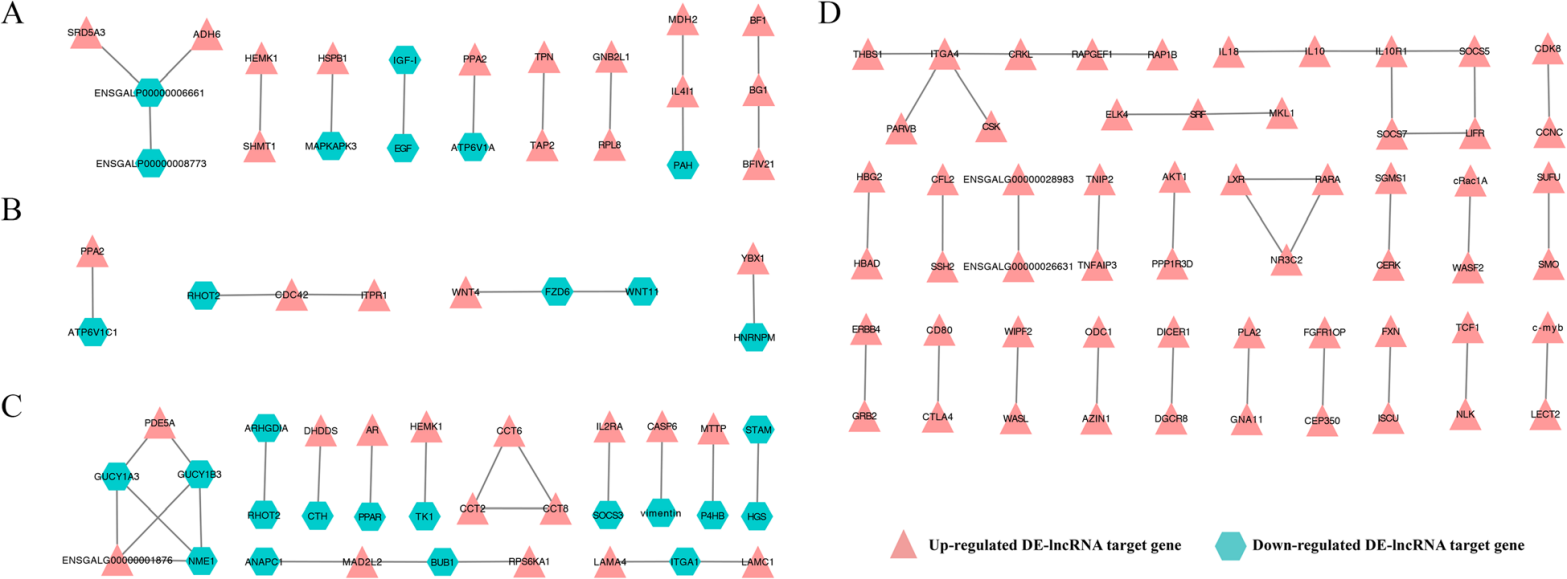
The PPI network of DE-lncRNA target genes. (**a**) Cis-target genes of the *W14* vs. *W6* comparison group; (**b**) Cis-target genes of the *W22* vs. *W14* comparison group; (**c**) Cis-target genes of the *W30* vs. *W22* comparison group; (D) Trans-target genes of the *W30* vs. *W22* comparison group

The PPI network was constructed by Cytoscape software using the predicted protein-protein interaction networks from the STRING database (Fig. 10). The PPI network from DE-lncRNA *cis*-target genes of the *W14* vs. *W6* comparison group contained 13 protein-protein pairs, such as *IGF-I-EGF*. Moreover, in the *W22* vs. *W14* comparison group, there were 6 protein-protein pairs, for example, *FZD6-WNT11*. Furthermore, the DEGs from the *W30* vs. *W22* comparison group included 23 protein-protein pairs, including *AR-PPAR*. However, no PPI network was found in the DE-lncRNA *trans*-target genes of the *W14* vs. *W6* and *W22* vs. *W14* comparison groups. In addition, the DE-lncRNA *trans*-targets from the *W30* vs. *W22* comparison group included 36 protein-protein pairs, including *CDK8-CCNC*.

## Discussion

The growth and development of skeletal muscle is regulated by many factors, such as nutrition, genetics, and the environment. In genetics research, previous studies focused on the role of protein-coding genes in skeletal muscle growth and development, and it was later found that noncoding RNA represents the majority of the transcriptome, with studies showing that only < 2% of mammalian genomes encode proteins [21]. The existence of a large number of noncoding RNAs must have value and significance. Noncoding RNAs, including miRNAs [22], lncRNAs [23, 24], and circRNAs [25], play different roles in complex biological processes. However, there are few studies on lncRNAs in chicken skeletal muscle development. In our study, we compared the differences in lncRNA expression in chicken breast muscles of chickens at four different ages (6, 14, 22, and 30 weeks) to identify important lncRNAs associated with muscle development.

Our research showed that chromosomal distribution of DE-lncRNAs was mainly concentrated in chromosome 1, which was similar to previous studies, i.e., there were more lncRNAs distributed in larger chromosomes [26]. The DE-lncRNAs on these chromosomes may exert their effects by regulating the expression of adjacent functional genes. Recent studies have demonstrated that lncRNAs can control the expression of important genes through *cis-* and *trans-*regulatory mechanisms [27]. In the present study, functional enrichment analyses of both *cis-* and *trans-*target genes of DE-lncRNAs revealed that the muscle development-related enriched GO terms were found only in the *W22* vs. *W14* comparison group. This is consistent with our previous transcriptomics analysis [19] and indicates that the period from 14 to 22 weeks is an important stage in the development of chicken breast muscle. In addition, in the pathway enrichment analysis of the DE-lncRNA *trans-*target genes, it was found that there was a common pathway in the *W22* vs. *W14* and *W30* vs. *W22* comparison groups, the MAPK signaling pathway. The MAPK family plays crucial roles in complex cellular processes, such as proliferation, differentiation, and development, by regulating the cell cycle and other cell proliferation-associated proteins [28]. We speculate that the DE-lncRNAs in the *W22* vs. *W14* and *W30* vs. *W22* comparison groups may play important roles in regulating muscle cell proliferation. Moreover, the function of lncRNAs can be inferred from associated contiguous or co-expressed protein-coding genes [29]. Thus, we constructed interaction networks of DE-lncRNAs and their *cis-* and *trans-*target genes, and only some of the genes associated with muscle development-related GO terms and the MAPK signaling pathway and their corresponding lncRNAs are shown in Fig. 6. Interestingly, we found that the networks containing *MEF2C* and its targeting lncRNAs were not only in the muscle development-related GO terms but also in the MAPK signaling pathway. Research has shown that *MEF2* factors are involved in muscle differentiation, and *MyoD* and *MEF2* family members interact to activate transcription and myogenesis [30]. Thus, we speculate that these lncRNAs, such as ALDBGALT0000008862, ALDBGALT0000008865, and LNC_001247, are involved in muscle differentiation.

LncRNAs have significant similarities to classic mRNAs during transcription, so miRNAs can not only target mRNAs but also regulate target lncRNAs and reduce their structural and functional stability [31]. It has been speculated that miRNA-192 and miRNA-204 directly suppress lncRNA HOTTIP and interrupt GLS1-mediated glutaminolysis in hepatocellular carcinoma [32]. Interestingly, miRNAs can also enhance lncRNA expression through several mechanisms [33]. miR-140 binding sites were identified in *NEAT1*, and the authors found that mature miR-140 could physically interact with *NEAT1* in the nucleus, thereby promoting the expression of *NEAT1* [34]. The predicted miRNA-lncRNA networks are shown in Fig. 7 and suggest that miRNAs may play a role in promoting or inhibiting lncRNA expression. Interestingly, several muscle-specific miRNAs, such as gga-miR-206, gga-miR-1a-3p, and miR-133a-3p, have been identified [35]. Therefore, we speculate that the target lncRNAs of these miRNAs may also be involved in the muscle development process. In addition, lncRNAs play many complex roles in gene expression and signal transduction and can also be used as precursors of miRNAs. LncRNAs can produce specific miRNAs through intracellular RNA splicing to affect the posttranscriptional regulation of mRNAs. It has been found that the H19 RNA is a miRNA precursor and generates the exonic microRNA miR-675 [36]. In this study, we found 5 pairs of homologous lncRNAs and pre-miRNAs, including ALDBGAL0000008009 with gga-miR-N1 and lnc_000010 and lnc_000011 with gga-miR-N2. We believe that the miRNAs produced by these lncRNAs play a functional role.

There is increasing evidence that supports the ceRNA hypothesis that lncRNAs regulate target genes by competitively adsorbing miRNAs [37]. For instance, lncRNA-Unigene56159 can directly bind to miR-140-5p and act as a ceRNA of miR-140-5p to regulate the expression of its target gene *Slug*, thereby affecting HCC cell migration and invasion [38]. Furthermore, lncRNA-MEG3 acts as a tumor suppressor and a ceRNA that regulates *E-cadherin* and forkhead box O1 (*FOXO1*) expression by competitively binding to miR-9 [39]. In thyroid cancer, lncRNA-Gas5 regulates phosphate and tension homology deleted on chromosome ten (*PTEN*) expression through a ceRNA mechanism as a sponge for miR-222-3p [40]. To fully identify how lncRNA-associated ceRNA networks affect breast muscle development in different developmental stages, we predicted and successfully constructed a network of ceRNAs associated with DE-lncRNAs in different comparison groups. For example, in the *W14* vs. *W6* comparison group, there were ceRNA networks containing the *DYNLL2* gene with 12 lncRNAs that targeted 2 miRNAs, gga-miR-148a-3p and gga-miR-130b-3p (Fig. 9a). *DYNLL2* is involved in a variety of cellular processes and interacts with myosin 5a (myo5a) to participate in cargo transport [41]. Therefore, we speculate that the above 12 lncRNAs may regulate *DYNLL2* by adsorbing miR-148a-3p and gga-miR-130b-3 to regulate muscle development. Furthermore, the ceRNA network analysis showed that *ANKRD1* is involved in 13 ceRNA networks containing two miRNAs (miR-148a-3p and miR-10b-5p) and 12 lncRNAs in the *W14* vs. *W6* comparison group (Fig. 9a). Moreover, *ANKRD1* was involved in 8 ceRNA networks containing gga-miR-92-3p with 8 lncRNAs in the *W30* vs. *W22* comparison group (Fig. 9c). Studies have shown that *ANKRD1* can induce gene expression in cultured skeletal muscle cells and trigger signaling via myogenic regulators (*MRFs*) during myogenesis [42]. Therefore, we think that the above 20 lncRNAs can also play roles in skeletal muscle cells to regulate the *ANKRD1* gene by adsorbing miR-148a-3p, miR-10b-5p, and gga-miR-126-5p.

In addition, we also demonstrated the interactions between DE-lncRNA target genes in Gushi chicken breast development. The interaction between *IGF-I* and epidermal growth factor (*EGF*) was identified in the *W14* vs. *W6* comparison group. It has been reported that the expression of the *IGF* gene is enhanced during muscle hypertrophy and that locally produced *IGF-I* may play roles in skeletal muscle growth [43]. Heparin-binding epidermal growth factor (*HB-EGF*) promotes airway smooth muscle (ASM) cell proliferation through the MAPK pathway [44]. Thus, we surmise that *IGF-I* and *EGF* can interact to play a role in skeletal muscle cell proliferation. Moreover, the PPI networks based on the *W22* vs. *W14* comparison group included *FZD6-WNT11* among others. The WNT/planar cell polarity (PCP) signaling pathway is involved in the development of human cancer. In this signaling pathway, winglesstype MMTV integration site family 11 (*WNT11*) can transduce PCP signals through receptors such as frizzled homolog 6 (*FZD6*) [45]. Thus, we speculate that *FZD6* and *WNT11* can interact to play a role in cell proliferation. In addition, some of the DE-lncRNA *trans*-targets from the *W30* vs. *W22* comparison group were included in the *CDK8-CCNC* PPI network. Research has shown that cyclin-dependent kinase 8 (*CDK8*) and cyclin C (*CCNC*) are transcriptional regulators that mediate several oncogenic pathways [46]. Therefore, the interactions between these genes may eventually affect muscle development, and the above results indicate that there are complex intergenic interactions in the development of chicken breast muscle.

## Conclusions

In conclusion, we first described the lncRNA profile of Gushi chicken breast muscle development at 6, 14, 22, and 30 weeks. In the *W22* vs. *W14* comparison groups, some GO terms related to muscle development were found, further indicating that between 14 and 22 weeks, changes in the expression of some crucial lncRNAs and their target genes affected the growth and development of chicken breast muscles during these important stages. In addition, the MAPK signaling pathway was found to play a vital role in muscle development. *MEF2C* and its target lncRNA, such as ALDBGALT0000008862, ALDBGALT0000008865, and LNC_001247, may be involved in muscle regulation through the MAPK signaling pathway. These results provide insight and valuable resources for further research on the molecular mechanisms of skeletal muscle development after hatching.

## Methods

### Animals and sample preparation

The experimental animals in this study were Gushi chickens from the Animal Center of Henan Agricultural University. A total of 300 one-day-old female Gushi chickens were raised in cages with the same environment, with standard conditions for pure breeding conservation of Gushi chickens. In this study, three healthy chickens were randomly selected at 6, 14, 22, and 30 weeks of age. Therefore, twelve chickens were used in this study. Our sample size was sufficient, and the remaining healthy chickens are still used for the pure breeding conservation of Gushi chickens. These chickens had a two-stage feeding protocol, in which 18.5% crude protein and 12.35 MJ/kg were prepared in the first stage (younger than 14 weeks) and 15.6% crude protein and 12.75 MJ/kg were prepared in the second stage (older than 14 weeks), and the chickens had free access to water. Chickens were anesthetized by intravenous injection of sodium pentobarbital (40 mg/kg) at a concentration of 0.2% in the wing vein. Under deep anesthesia, these individuals were euthanized by intravenous KCL (1–2 mg/kg). The left breast muscle tissue was then collected, immediately frozen in liquid nitrogen and stored at − 80 °C until RNA extraction.

### Illumina deep sequencing and sequence analysis

Twelve RNA libraries were constructed using 12 breast muscle samples (W6, W14, W22, and W30; each stage had three individual samples). Total RNA was isolated from breast muscle samples using TRIzol reagent (Invitrogen, Carlsbad, CA, USA) following the manufacturer’s instructions. The quantity and quality of RNA were evaluated by NanoDrop 2000 (Thermo Scientific, Wilmington, DE, USA) and by 1% agarose gel electrophoresis. A total of 3 μg RNA per sample was used as input material for the cDNA library. First, the Epicentre Ribo-zero™ rRNA Removal Kit (Epicentre, Madison, Wisconsin, USA) was used to remove ribosomal RNA. Second, sequencing libraries were generated using rRNA-depleted RNA with the NEBNext® Ultra™ Directional RNA Library Prep Kit for Illumina® (NEB, Ipswich, MA, USA) following the manufacturer’s instructions. The products were then purified using a TruSeq RNASample Prep Kit v2 (New England Biolabs, Ipswich, MA, USA), and library quality was assessed on an Agilent Bioanalyzer 2100 system (Agilent Technologies, CA, USA). Finally, the libraries were sequenced using an Illumina HiSeq 2500 platform, and paired-end reads were generated. The raw data in the fastq format were first processed by in-house scripts. The Illumina sequencing raw reads were obtained by removing adapter sequences, reads with poly-N and low-quality reads, in which the number of bases with a quality value Q ≤ 20 was > 50%. All downstream analyses were based on high-quality clean data. Reference genome and gene model annotation files were downloaded from a genome website (ftp://ftp.ensembl.org/pub/release-83/fasta/gallus_gallus/dna/). An index of the reference genome was built using Bowtie v2.2.3, and paired-end clean reads were aligned to the reference genome using TopHat v2.0.12. The Cufflinks v2.1.1 Reference Annotation Based Transcript (RABT) assembly method was used to construct and identify transcripts from the TopHat alignment results.

### Identification of lncRNAs

The combined transcript sets were screened for lncRNAs, and the lncRNA screening process was divided into the following five steps: the first step was to select the transcripts with exon number ≥ 2; the second step was to select the transcripts with transcript length > 200 bp; the third step was to use Cuffcompare software to filter out the transcripts that overlapped with exon regions annotated in the database, and the lncRNAs in the database that overlapped with the exon regions of splicing transcripts were annotated as lncRNAs in subsequent analyses; the fourth step was to calculate the expression of each transcript by Cuffquant, whereby transcripts with an FPKM (fragments per kilobase of exons per million mapped fragments) ≥ 0.5 expression level were selected; and the fifth step was to screen the protein-coding potential, in which the four databases CPC, PFAM, PhyloCSF and CNCI were used to remove potential protein-coding transcripts. In this study, the resulting transcripts with no protein-coding potential in the software analyses resulted in the lncRNA dataset, and the transcripts that were predicted to have protein-coding potential by at least one coding potential prediction software were set as TUCPs.

### Differential expression analysis

Cuffdiff (v2.1.1) was used to calculate the FPKM of lncRNAs in each sample, and cuffdiff used a model based on a negative binomial distribution to provide statistical procedures for determining differential expression in digital transcript or gene expression data [20]. Based on Illumina sequencing data, FPKM values were used to assess the expression levels of lncRNAs in the libraries constructed from breast muscle. The FC for each lncRNA between two discretionary stages was calculated according to comparisons between four comparison groups (W6, W14, W22, and W30). Differential expression analysis among the four groups was performed using the DESeq R package (1.8.3), and lncRNAs and genes found by DESeq with a *q-*value < 0.05 and |FC|≥1.7 were considered to be differentially expressed.

### *Cis*- and *trans*-targeting analyses

Differentially expressed lncRNAs were selected for *cis*- and *trans*-target gene predictions and were integrated with differentially expressed gene data to improve the veracity of target prediction. In the present study, DEGs located ∼100 kb upstream and downstream of DE-lncRNAs were classified as *cis-*acting target genes. In addition, we predicted the regulation in *trans* between lncRNAs and genes by the Pearson correlation coefficient, r > 0.95. The relationship between lncRNAs and their target genes was demonstrated by Cytoscape 3.4 (http://www.cytoscape. org/).

All potential target genes for DE-lncRNAs in each comparison group were used in the bioinformatics analysis. GO and KEGG pathway analyses were conducted using the DAVID database [47] to visualize data. Only GO terms and KEGG pathways with corrected *p*-values (*t*-test) < 0.05 were considered significantly enriched.

### Association analysis and interaction network construction

The data including mRNAs [3] and miRNAs [19] obtained from our previous study were used for integrative analysis with the lncRNA data. The transcriptome and small RNA library were constructed using the same tissue RNA Gushi chicken breast muscle samples from chickens at the four developmental stages in our previous research. Additionally, a *q-*value < 0.05 was set as the threshold for significant DEMs by default. DEMs, DEGs, and DE-lncRNAs were used to construct lncRNA-miRNA-mRNA interaction networks by Cytoscape 3.4. PPI analysis of DEGs targeted by DE-lncRNAs was based on the STRING database (https://string-db.org, Organism: *Gallus gallus*). The network of interactions between these DEGs was generated in the STRING database and imported into Cytoscape software for visualization; scores > 700 were selected as significant interactions to display.

### Quantitative real-time PCR (qRT-PCR) analysis

The qRT-PCR analysis of lncRNAs was performed using the PrimeScript™ RT reagent kit (TaKaRa, Dalian, China) according to the manufacturer’s instructions. qRT-PCR was performed with a LightCycler® 96 instrument qRT-PCR system (Roche, Basel, Switzerland) with the PrimeScript™ RT Reagent Kit with gDNA Eraser (Takara, Kyoto, Japan). The amplification program consisted of 95 °C for 3 min; 40 cycles of 95 °C for 10 s; and annealing at 60 °C for 30 s, 72 °C for 30 s, and 72 °C for 10 min. The relative expression levels were analyzed with the 2^−△△Ct^ method. The qRT-PCR primer sequences are listed in Table S6.

### Statistical analysis

The qRT-PCR quantitative expression data and graphs were generated in GraphPad Prism (version 5.0) software (San Diego, CA, USA). Statistical significance of the qRT-PCR quantitative expression data was tested by performing two-tailed unpaired t-tests [48]. Data are presented as the means containing three replicates.

## Supplementary Information

**Additional file 1: Fig. S1.** Heatmap showing DE-lncRNAs from different stages.

**Additional file 2: Fig. S2.** The chromosome distribution of DE-lncRNAs from different stages.

**Additional file 3: Fig. S3.** The enriched GO terms of the DE-lncRNA. (A-B) Cis-target genes in the *W14* vs. *W6* and *W30* vs. *W22* comparison groups. (C-D) Trans-target genes in the *W14* vs. *W6* and *W30* vs. *W22* comparison groups.

**Additional file 4: Fig. S4**. The enriched KEGG pathways of the DE-lncRNA. (A-C) Cis-target genes in the *W14* vs. *W6*, *W22* vs. *W14*, and *W30* vs. *W22* comparison groups. (D) Trans-target genes in the *W14* vs. *W6* comparison groups.

**Additional file 5: Fig. S5**. The lncRNA-miRNA-mRNA ceRNA networks. (A) *W14* vs. *W6* comparison group; (B) *W22* vs. *W14* comparison group; (C) *W30* vs. *W22* comparison group.

**Additional file 6; Table S1**. Summary of draft reads of 12 cDNA libraries, determined by RNA sequencing. Abbreviations: W6_1, sample 1 of 6 weeks; W6_2, sample 2 of 6 weeks; W6_3, sample 3 of 6 weeks; W14_1, sample 1 of 14 weeks; W14_2, sample 2 of 14 weeks; W14_3, sample 3 of 14 weeks; W22_1, sample 1 of 22 weeks; W22_2, sample 2 of 22 weeks; W22_3, sample 3 of 22 weeks; W30_1, sample 1 of 30 weeks; W30_2, sample 2 of 30 weeks; W30_3, sample 3 of 30 weeks.

**Additional file 7: Table S2**. Cis-regulatory interactions between DE-lncRNAs and DE-mRNAs.

**Additional file 8: Table S3**. Trans-regulatory interactions between DE-lncRNAs and DE-mRNAs.

**Additional file 9: Table S4**. The interaction between DE-lncRNAs and DE-miRNAs.

**Additional file 10: Table S5**. Details on the novel miRNAs identified in this study. Abbreviations: W6, W14, W22, and W30 represent small RNA libraries obtained using samples from chickens aged 6, 14, 22, and 30 weeks, respectively.

**Additional file 11: Table S6**. qRT-PCR primers. Abbreviation: AT refers to the annealing temperature; F and R refer to the forward and reverse primers, respectively.

## Data Availability

All raw sequences have been deposited in the NCBI database Sequence Read Archive with the accession numbers PRJNA516810 (mRNA and lncRNA) and PRJNA516961 (miRNA).

## Abbreviations

GHSR: Growth hormone secretagogue receptor
IGFs: Insulin-like growth factors
TGFβ2: Transforming growth factor beta 2
MEF2B: Myocyte enhancer factor 2B
lncRNAs: Long noncoding RNAs
MyoD: Myogenic differentiation antigen
IRS1: Insulin receptor substrate 1
TUCPs: Transcripts of uncertain coding potential
lincRNA: Long intergenic noncoding RNAs
DE-lncRNAs: Differentially expressed lncRNAs
W6: 6 Weeks
DEG: Differentially expressed gene
GO: Gene ontology
KEGG: Kyoto encyclopedia of genes and genomes
DEMs: Differentially expressed miRNAs
ANKRD1: Ankyrin repeat domain 1
DYNLL2: Dynein light chain 2
MYH11: Myosin heavy polypeptide 11
RCAN1: Regulator of calcineurin 1
FOXO1: Forkhead box O1
PTEN: Phosphate and tension homology deleted on chromosome ten
MRFs: Myogenic regulators
EGF: Epidermal growth factor
HB-EGF: Heparin-binding epidermal growth factor
ASM: Airway smooth muscle
PCP: Planar cell polarity
WNT11: Winglesstype MMTV integration site family 11
FZD6: Frizzled homolog 6
CDK8: Cyclin-dependent kinase 8
CCNC: Cyclin C
