# Supplementary figures and images for: LncRNAs and their regulatory networks in breast muscle tissue of Chinese Gushi chickens during late postnatal development
Source: BMC Genomics. 2021 Jan 9;22:44. doi: 10.1186/s12864-020-07356-6 (PMC7797159; doi:10.1186/s12864-020-07356-6)

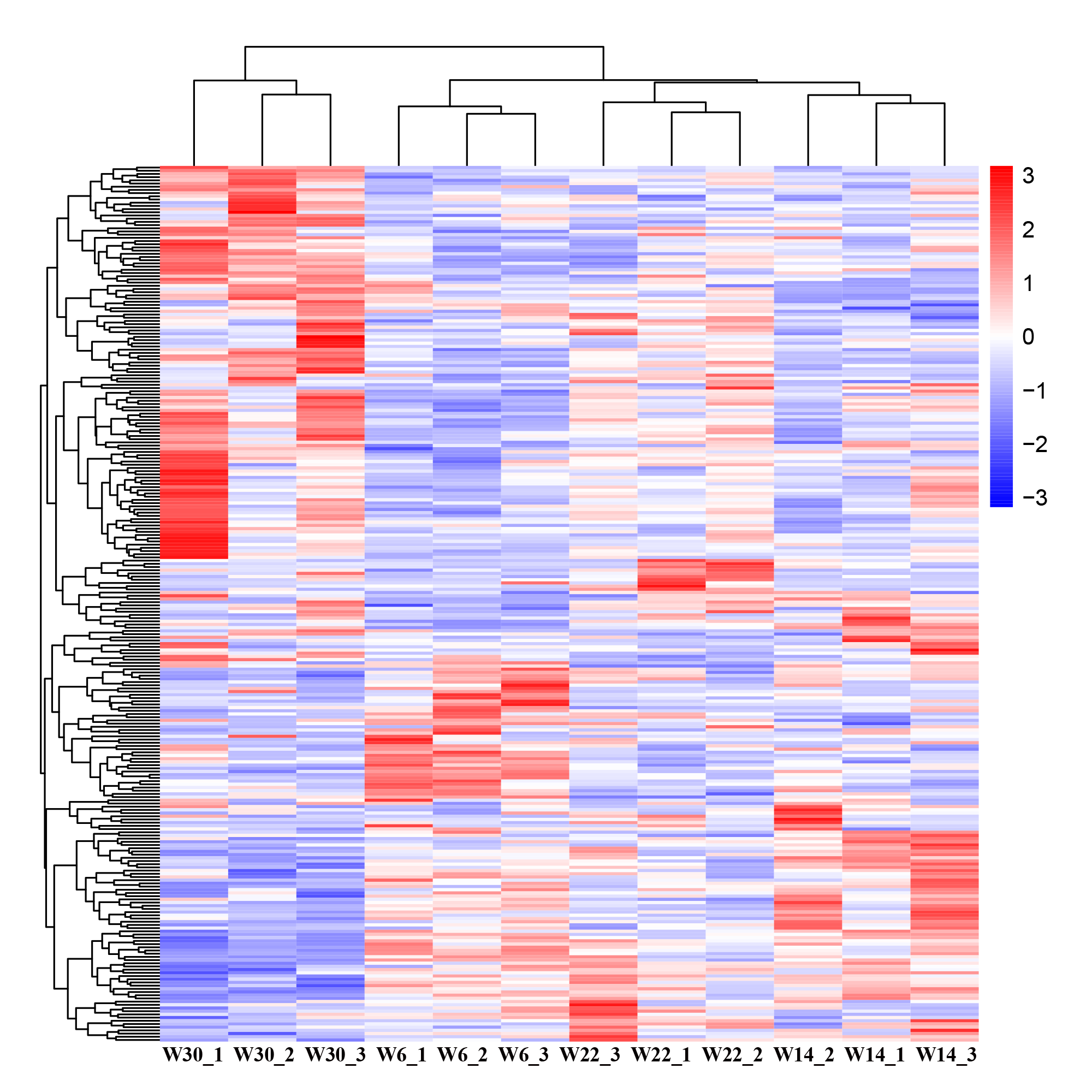

Supplement: Supplementary file 1 — Additional file 1: Fig. S1. Heatmap showing DE-lncRNAs from different stages. [file 12864_2020_7356_MOESM1_ESM.tif]

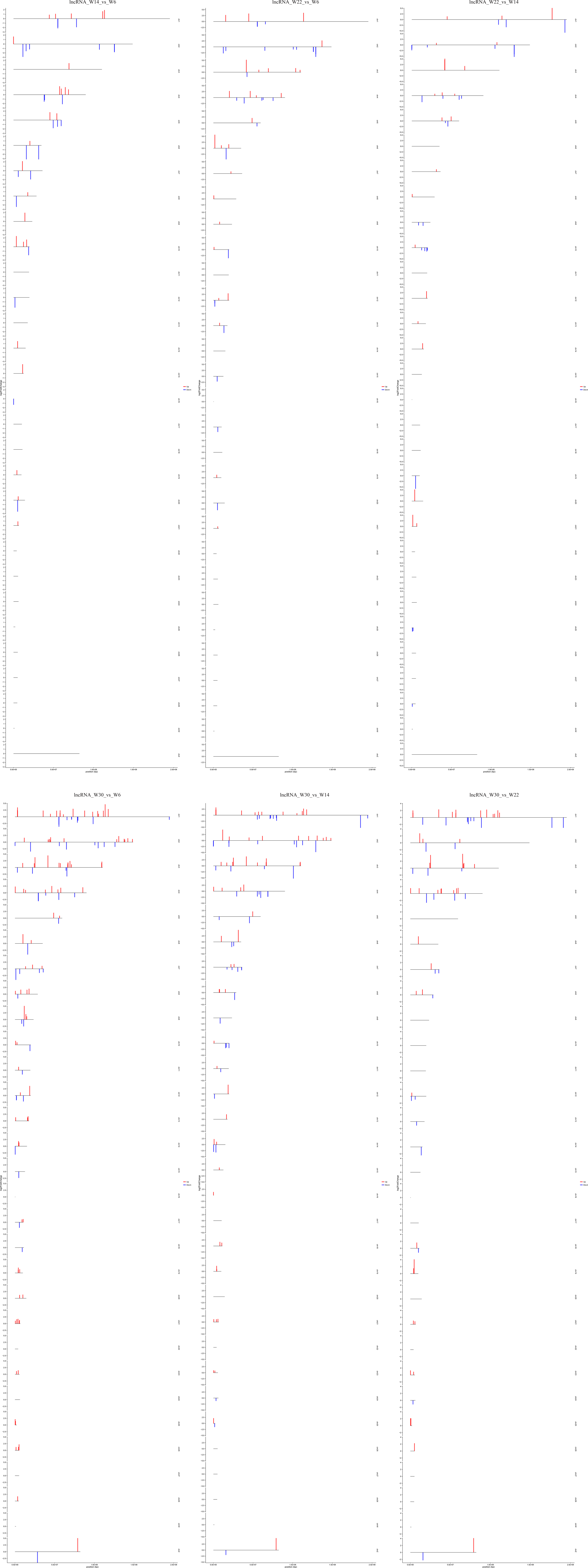

Supplement: Supplementary file 2 — Additional file 2: Fig. S2. The chromosome distribution of DE-lncRNAs from different stages. [file 12864_2020_7356_MOESM2_ESM.tif]

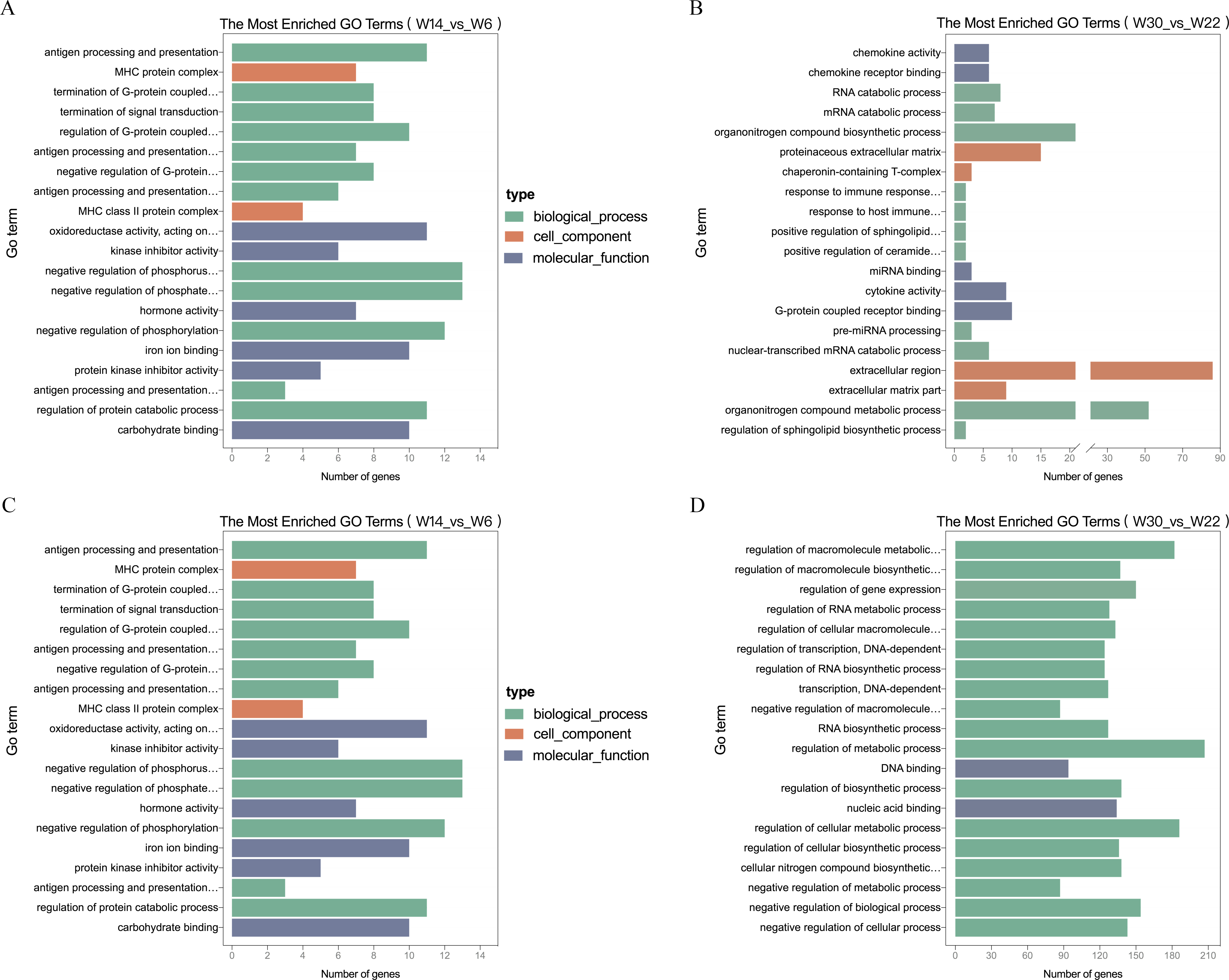

Supplement: Supplementary file 3 — Additional file 3: Fig. S3. The enriched GO terms of the DE-lncRNA. (A-B) Cis-target genes in the W14 vs. W6 and W30 vs. W22 comparison groups. (C-D) Trans-target genes in the W14 vs. W6 and W30 vs. W22 comparison groups. [file 12864_2020_7356_MOESM3_ESM.tif]

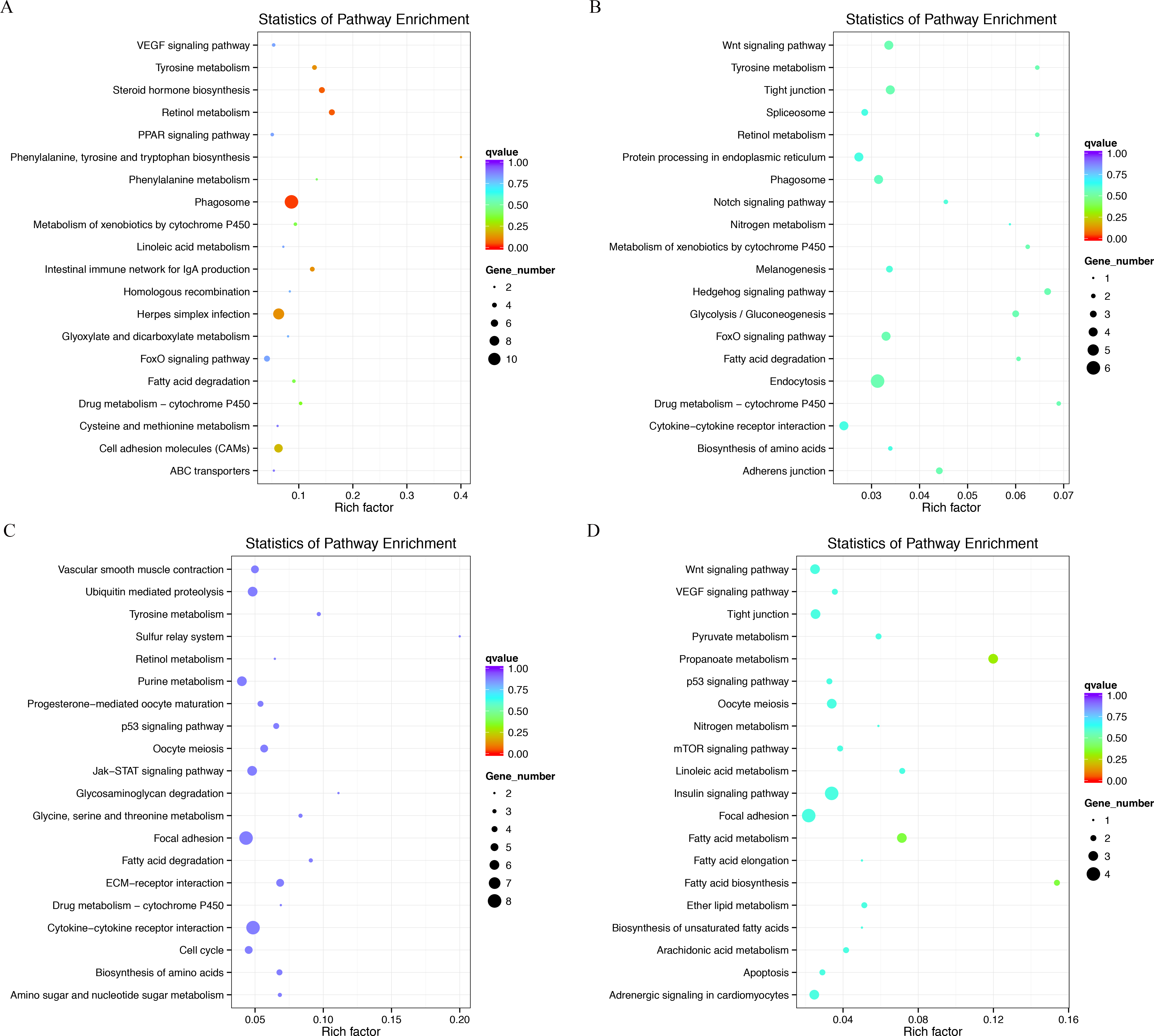

Supplement: Supplementary file 4 — Additional file 4: Fig. S4. The enriched KEGG pathways of the DE-lncRNA. (A-C) Cis-target genes in the W14 vs. W6, W22 vs. W14, and W30 vs. W22 comparison groups. (D) Trans-target genes in the W14 vs. W6 comparison groups. [file 12864_2020_7356_MOESM4_ESM.tif]

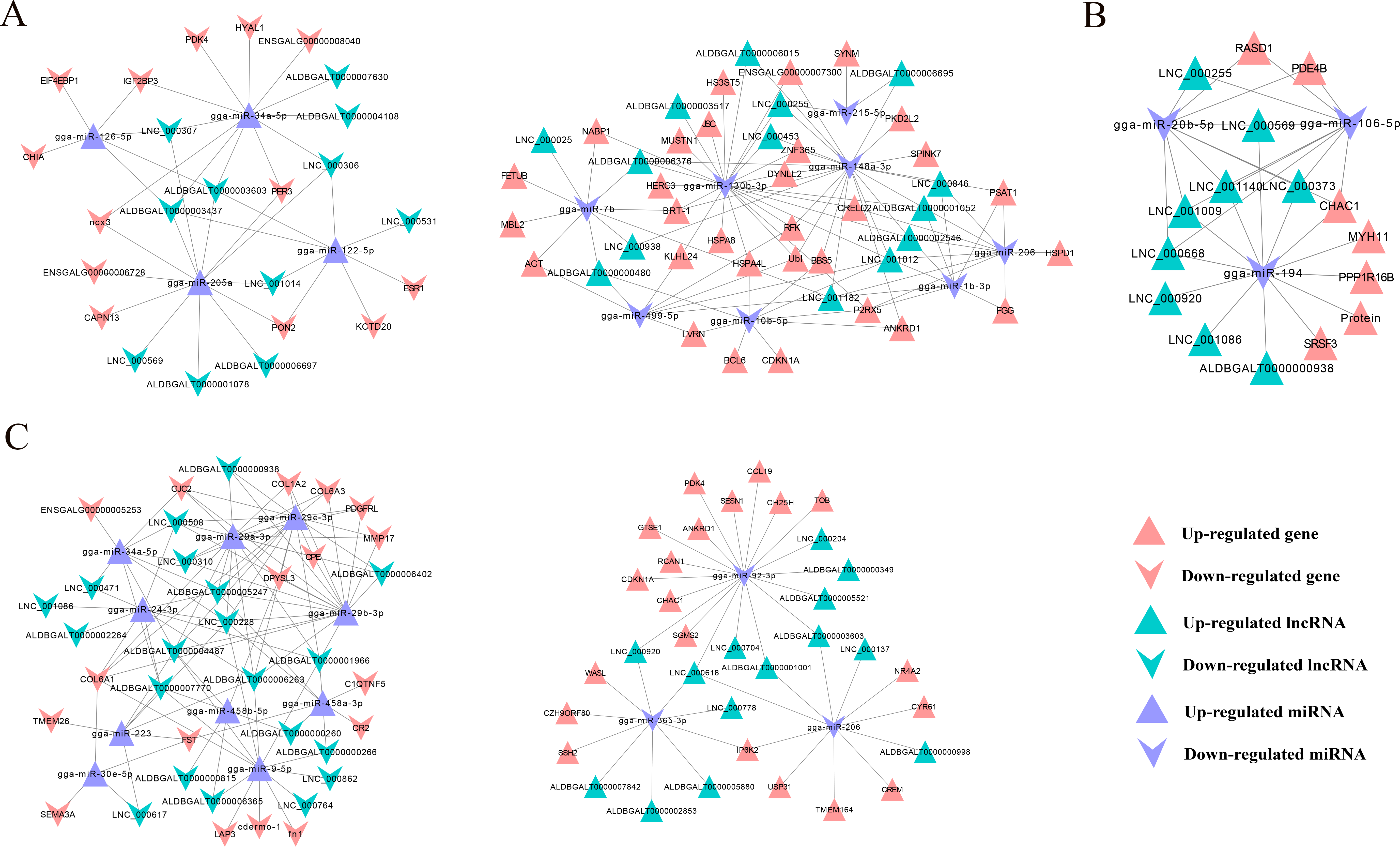

Supplement: Supplementary file 5 — Additional file 5: Fig. S5. The lncRNA-miRNA-mRNA ceRNA networks. (A) W14 vs. W6 comparison group; (B) W22 vs. W14 comparison group; (C) W30 vs. W22 comparison group. [file 12864_2020_7356_MOESM5_ESM.tif]
