# Supplementary material for: LncRNAs and their regulatory networks in breast muscle tissue of Chinese Gushi chickens during late postnatal development
Source: BMC Genomics. 2021 Jan 9;22:44. doi: 10.1186/s12864-020-07356-6 (PMC7797159; doi:10.1186/s12864-020-07356-6)
Supplement: Supplementary file 6 — Additional file 6; Table S1. Summary of draft reads of 12 cDNA libraries, determined by RNA sequencing. Abbreviations: W6_1, sample 1 of 6 weeks; W6_2, sample 2 of 6 weeks; W6_3, sample 3 of 6 weeks; W14_1, sample 1 of 14 weeks; W14_2, sample 2 of 14 weeks; W14_3, sample 3 of 14 weeks; W22_1, sample 1 of 22 weeks; W22_2, sample 2 of 22 weeks; W22_3, sample 3 of 22 weeks; W30_1, sample 1 of 30 weeks; W30_2, sample 2 of 30 weeks; W30_3, sample 3 of 30 weeks. [file 12864_2020_7356_MOESM6_ESM.docx]

**Table S1. Summary of draft reads of 12 cDNA libraries, determined by RNA-sequencing**

| Library name | Raw reads | Clean bases | Error rate(%) |
| --- | --- | --- | --- |
| W6_1 | 105,872,366 | 15.27G | 0.01 |
| W6_2 | 98,889,740 | 14.11G | 0.01 |
| W6_3 | 100,815,554 | 14.35G | 0.01 |
| W14_1 | 117,197,064 | 16.66G | 0.01 |
| W14_2 | 98,255,520 | 14.04G | 0.01 |
| W14_3 | 89,496,872 | 12.75G | 0.01 |
| W22_1 | 101,944,936 | 14.48G | 0.02 |
| W22_2 | 96,278,608 | 13.68G | 0.01 |
| W22_3 | 114,606,992 | 16.35G | 0.01 |
| W30_1 | 106,845,354 | 15.47G | 0.02 |
| W30_2 | 89,750,270 | 12.88G | 0.02 |
| W30_3 | 96,537,600 | 13.89G | 0.02 |

Abbreviations: W6_1, sample 1 of 6 weeks; W6_2, sample 2 of 6 weeks; W6_3, sample 3 of 6 weeks; W14_1, sample 1 of 14 weeks; W14_2, sample 2 of 14 weeks; W14_3, sample 3 of 14 weeks; W22_1, sample 1 of 22 weeks; W22_2, sample 2 of 22 weeks; W22_3, sample 3 of 22 weeks; W30_1, sample 1 of 30 weeks; W30_2, sample 2 of 30 weeks; W30_3, sample 3 of 30 weeks.
