# Supplementary material for: LncRNAs and their regulatory networks in breast muscle tissue of Chinese Gushi chickens during late postnatal development
Source: BMC Genomics. 2021 Jan 9;22:44. doi: 10.1186/s12864-020-07356-6 (PMC7797159; doi:10.1186/s12864-020-07356-6)
Supplement: Supplementary file 10 — Additional file 10: Table S5. Details on the novel miRNAs identified in this study. Abbreviations: W6, W14, W22, and W30 represent small RNA libraries obtained using samples from chickens aged 6, 14, 22, and 30 weeks, respectively. [file 12864_2020_7356_MOESM10_ESM.docx]

**Table S5.** Details on the novel miRNAs identified in this study

| miRNA ID | Mature miRNA sequence | sRNA read count | | | | Precursor sequence | Genome localization |
| --- | --- | --- | --- | --- | --- | --- | --- |
|  |  | W6 | W14 | W22 | W30 |  |  |
| >gga-miR-N1 | uucuccucccuucgcacggcgca | 0 | 0 | 0 | 4 | ugccggggcgcuggggaggggcagcuccccuuugcugcccugcuucuccucccuucgcacggcgca | 8:27165844..27165956:- |
| >gga-miR-N2 | aucaccaaggcugggugcuguga | 0 | 0 | 2 | 1 | aucaccaaggcugggugcugugagagccacaugcagaucggagcagccagcuuugggaugg | AADN03010814.1:1659..1771:+ |
| >gga-miR-N3 | uuucccuuuggauuuuucucucuu | 4 | 1 | 3 | 0 | uuucccuuuggauuuuucucucuugcuguaugcuuaaaacaagaggaagggagaaugaagggaaaag | 1:35702625..35702738:+ |
| >gga-miR-N4 | agcguucugcuuuccuggugug | 1 | 1 | 1 | 1 | agcguucugcuuuccugguguggugcuggaguuucccuuacacgagcagagcagggcgcugg | 4:85065528..85065638:- |

Abbreviations: W6, W14, W22, and W30 represent small RNA libraries obtained using samples from chickens aged 6, 14, 22, and 30 weeks, respectively.
