# Supplementary material for: LncRNAs and their regulatory networks in breast muscle tissue of Chinese Gushi chickens during late postnatal development
Source: BMC Genomics. 2021 Jan 9;22:44. doi: 10.1186/s12864-020-07356-6 (PMC7797159; doi:10.1186/s12864-020-07356-6)
Supplement: Supplementary file 11 — Additional file 11: Table S6. qRT-PCR primers. Abbreviation: AT refers to the annealing temperature; F and R refer to the forward and reverse primers, respectively. [file 12864_2020_7356_MOESM11_ESM.docx]

**Table S6. qRT-PCR primers.**

| **Target** | **Primer** | **Sequence（5’-3’）** |  | **AT(**°**C)** |
| --- | --- | --- | --- | --- |
| Lnc_000037 | F | ACCCGGTGATGACCGACTTA |  | 60 |
|  | R | CACACCCCTTCAGAGCACAT |  |  |
| ALDBGALT0000002856 | F | CAGCAGCTCTCTATGAGCCC |  | 60 |
|  | R | GGCACCTTTTCACATGCCAG |  |  |
| ALDBGALT0000004123 | F | GAACATCATCCCAGCGTCCA |  | 60 |
|  | R | GAACATCATCCCAGCGTCCA |  |  |
| ALDBGALT0000007927 | F | CAGCAGCTCTCTATGAGCCC |  | 60 |
|  | R | GGCACCTTTTCACATGCCAG |  |  |
| GAPDH | F | GAACATCATCCCAGCGTCCA |  | 60 |
|  | R | GAACATCATCCCAGCGTCCA |  |  |

Abbreviation: AT refers to the annealing temperature; F and R refer to the forward and reverse primers, respectively.
